# Enhancing Oxygen Evolution Reaction at High Current Densities on Amorphous‐Like Ni–Fe–S Ultrathin Nanosheets via Oxygen Incorporation and Electrochemical Tuning

**DOI:** 10.1002/advs.201600343

**Published:** 2016-12-20

**Authors:** Jingfang Zhang, Yuchen Hu, Dali Liu, Yu Yu, Bin Zhang

**Affiliations:** ^1^ Department of ChemistrySchool of Science and Tianjin Key Laboratory of Molecular Optoelectronic ScienceTianjin UniversityTianjin300072China; ^2^ Collaborative Innovation Center of Chemical Science and EngineeringTianjin300072China

**Keywords:** amorphous, electrochemical tuning, high current densities, oxygen evolution, ultrathin nanosheets

## Abstract

**Amorphous‐like Ni–Fe–S ultrathin nanosheets by oxygen incorporation and electrochemical tuning** show excellent OER activity at high current densities. Such excellent performance could be attributed to the unique 3D porous configuration composed of amorphous‐like ultrathin nanosheets with much more active sites and improved conductivity, as well as the proper electronic structure benefiting from the oxygen incorporation and electrochemical tuning.

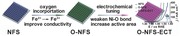

Great expectations have been held for electrolytic water splitting technologies with potential renewable and sustainable energy source to meet the rising energy demands.^1^ As a half reaction process in water electrolysis, the oxygen evolution reaction (OER) severely suffers from the sluggish kinetics and thus restricts the overall efficiency of energy conversion.^2^ To date, RuO_2_ and IrO_2_ are considered as the most active electrocatalysts for OER.^3^ Unfortunately, their widespread applications are seriously confined by the high price and scarcity of these noble‐metal oxides. Also, they are usually adhered to the electrode surface with a polymer binder for electrocatalysis, which generally results in the blockage of active sites and falling off of samples from the electrode with vigorous bubbles attacking during time‐consuming electrolysis process.^4^ Hence, it is desirable but challenging to explore earth‐abundant, highly active and robust electrocatalysts tightly immobilized on conductive substrates for OER, especially under large current densities (>500 mA cm^−2^).

Transition‐metal (e.g., Ni, Co, Fe) based materials, including oxides,^5^ (oxy)hydroxides,[[qv: 3a,6]] selenides,^7^ sulfides,^8^ and nitrides,^9^ have been extensively investigated as promising candidates of high‐performance catalysts for OER. Despite these impressive progress in OER activity, it is still a long way from industrial applications with requirement of very large current densities at low overpotentials.[[qv: 2a]] Recent works have demonstrated the electroactivity mainly depends on the surface active sites and electronic structure of the electrocatalysts.[[qv: 5a,10]] Compared to high crystalline materials, the corresponding amorphous ones own more unsaturated atoms as active sites and thus enhanced catalytic activity.^11^ On the other hand, the surface electronic structure could be modulated by element incorporation.[[qv: 10c,12]] As an example, Fe impurity is certified to greatly improve the OER performance of nickel‐base catalysts.^13^ In addition, electrochemical tuning (ECT) can be considered as an effective approach to modify the electronic structure of metal and thus tune its bond energy as well as increase active area, leading to enhanced catalytic activity.[[qv: 1h,8b]] However, the rational design of highly efficient amorphous‐like OER electrocatalysts via element incorporation, especially combining with ECT strategy, still remains a huge challenge.

Herein, we present a new approach to synthesize amorphous‐like oxygen‐incorporated Ni–Fe–S (NFS) ultrathin nanosheets supported on Ti plate (denoted as O‐NFS) through a facile calcination of Ni–Fe–S ultrathin nanosheets on Ti plate (denoted as NFS) in air, followed by ECT as an efficient OER electrocatalyst (**Figure** **1**a). The oxygen incorporation and ECT can be conductive to increase electric conductivity and modulate electronic structure as well as the active sites. The resulted product is a highly active and stable electrocatalyst for OER at large current densities up to 3000 mA cm^−2^.

**Figure 1:**
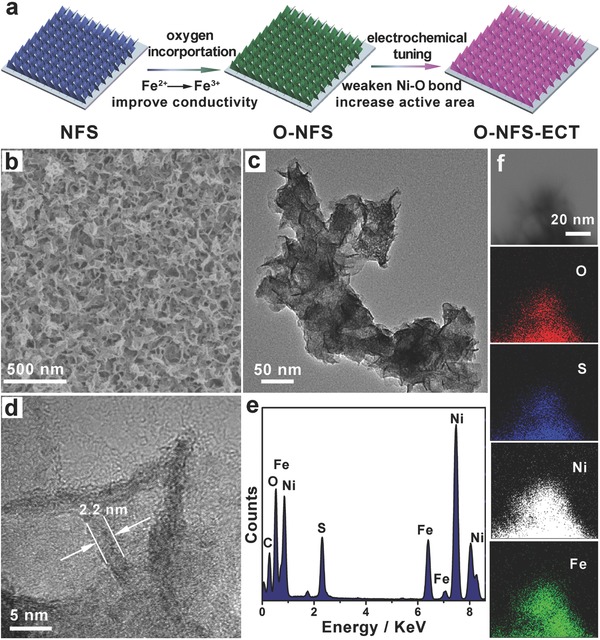
a) Schematic illustration of the preparation of O‐NFS ultrathin nanosheets. b) Representative SEM image, c) TEM image, d) HRTEM image, e) EDS spectrum, and f) STEM‐EDS mapping images of the O‐NFS ultrathin nanosheets.

The NFS ultrathin nanosheets are synthesized via an electrodeposition method from aqueous solution containing nickel salt, iron salt, and thiourea (see the Experimental Section for details). The as‐prepared samples are first characterized by scanning electron microscopy (SEM). As shown in Figure S1a,b in the Supporting Information, the nanosheet arrays are grown tightly on Ti plate on a large scale. The interconnected nanosheets construct a 3D open structure, facilitating effectively the electrolyte penetration and charge transport.^14^ The low‐magnification transmission electron microscopy (TEM) image further demonstrates the porous nanosheet array structure (Figure S1c, Supporting Information). The high‐resolution TEM (HRTEM, Figure S1d, Supporting Information) suggests an ultrathin thickness (2–3 nm) of the NFS nanosheets. Moreover, the HRTEM image shows ambiguous or invisible lattice fringes in the NFS ultrathin nanosheets, indicating the amorphous‐like nature of NFS ultrathin nanosheets. X‐ray photoelectron spectra (XPS; Figure S2, Supporting Information) and Raman (see below) analysis further signify the successful synthesis of NFS ultrathin nanosheets. The molar ratio of Fe, Ni, and S in NFS ultrathin nanosheets is close to 1:2.46:2.16.

The O‐NFS ultrathin nanosheets are fabricated by annealing the NFS ultrathin nanosheets at 200 °C in air. The corresponding SEM and TEM images (Figure 1b,c) indicate that the O‐NFS maintains the original 3D open structure composed of ultrathin nanosheets. The ultrathin thickness of nanosheet is measured as ≈2.2 nm by HRTEM image (Figure 1d). In addition, no obvious lattice fringes and diffraction peaks are observed in HRTEM image (Figure 1d) and X‐ray diffraction (XRD) pattern (Figure S3, Supporting Information). It suggests the O‐NFS ultrathin nanosheets possess amorphous‐like structure. The unique nanosheets with ultrathin and amorphous nature can provide more exposure of low‐coordinated surface atoms and thus abundant catalytically active sites.[[qv: 7b,8a,15]] The energy dispersive X‐ray spectroscopy (EDS) analysis (Figure 1e) certifies the existence of Ni, Fe, O, and S elements. The scanning transmission electron microscopy (STEM)–EDS elemental mapping images (Figure 1f) of O‐NFS ultrathin nanosheets show that the Ni, Fe, S, and O elements are uniformly distributed throughout the whole nanosheets, suggesting the successful transformation from NFS to O‐NFS ultrathin nanosheets. Furthermore, Raman spectra (**Figure** **2**a) show the appearance of vibrational mode for Fe—O bond.^16^ (≈220 cm^−1^, marked with blue dotted box) as well as the vibrational modes of NFS in O‐NFS ultrathin nanosheets, revealing the incorporation of oxygen into NFS ultrathin nanosheets. Moreover, the oxygen atom bonded to metal in O‐NFS can also be testified by the comparison of O 1*s* XPS in O‐NFS and NFS, as well as the Ni 2*p*, Fe 2*p*, and S 2*p* regions of O‐NFS ultrathin nanosheets (Figure 2b and Figure S4, Supporting Information). The O 1*s* peaks located at 532.7 and 531.3 eV in O‐NFS and NFS ultrathin nanosheets can be attributed to the hydroxy group and adsorbed water on metal surface, respectively.^17^ The peak position at 529.9 eV in O‐NFS is corresponding to the binding energy of oxygen atom and metal,^17^ suggesting the existence of metal‐oxygen bonds. Similar phenomenon was observed in oxygen‐incorporated MoS_2_ nanomaterials.^18^ In addition, the oxygen amount in the form of metal‐oxygen bonds is estimated to 3.3% in O‐NFS based on the XPS analysis. Besides, the valence state of Fe exists as Fe^3+^ in the O‐NFS ultrathin nanosheets instead of the coexistence of Fe^2+^ and Fe^3+^ in the NFS ultrathin nanosheets. The XPS difference between NFS and O‐NFS ultrathin nanosheets suggests the different electronic structures. All the results distinctly prove successful synthesis of amorphous‐like O‐NFS ultrathin nanosheets.

**Figure 2:**
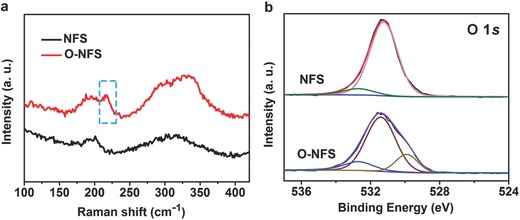
a) Raman spectra and b) O 1*s* XPS spectra for the as‐prepared O‐NFS and NFS ultrathin nanosheets.

To get insight into the influence of oxygen‐incorporation on the electrochemical performance toward OER, NFS, and O‐NFS ultrathin nanosheets are directly served as working electrodes to be tested in 1 m KOH solution at a scan rate of 10 mV s^−1^. Polarization curves of NFS and O‐NFS ultrathin nanosheets are recorded relative to the reversible hydrogen electrode (RHE). All the linear sweep voltammetry (LSV) curves are 95% *I*−*R* corrected. In addition, the OER activities of Ni–S and Fe–S samples are also tested to compare with NFS ultrathin nanosheets (Figure S5, Supporting Information). The NFS ultrathin nanosheets catalyst shows smaller overpotentials under the same current densities than that of Ni–S and Fe–S counterparts, indicating the better OER activity of NFS. As shown in **Figure** **3**a, the O‐NFS and NFS ultrathin nanosheets provide a current density of 200 mA cm^−2^ under overpotentials of 312 and 334 mV, respectively. In brief, the O‐NFS ultrathin nanosheets catalyst shows smaller overpotentials under the same current densities than that of NFS counterpart, indicating the better OER activity of O‐NFS. When the oxygen‐incorporation amount is further increased to 5.5% by annealing the NFS ultrathin nanosheets in air for 5 h (denoted as O‐NFS‐con), the current densities of O‐NFS‐con toward OER decrease compared with that of O‐NFS, indicating the proper oxygen‐incorporation amount is important for the OER activity (Figure S6, Supporting Information). From Nyquist plots of O‐NFS and NFS in Figure 3b, the O‐NFS catalyst shows a smaller charge transfer resistance than that of NFS, revealing the improved conductivity of O‐NFS due to the oxygen incorporation. The conductivity improvement implies that the electronic structure of O‐NFS has changed after the oxygen incorporation.^18^ In addition, the higher valence state of Fe in O‐NFS may make it harder to oxidize surrounding Ni, leading to the faster OER kinetics and enhanced OER activity.^13^, ^19^ Based on the above results, we can safely draw a conclusion that the suitable electronic structure of O‐NFS is responsible to the enhanced OER performance.

**Figure 3:**
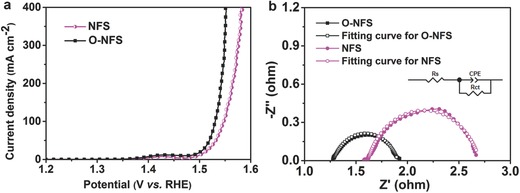
a) LSV curves and b) Nyquist plots at 1.576 V (vs RHE), the corresponding equivalent circuit (inset) and the equivalent‐circuit derived fitting plots for NFS and O‐NFS ultrathin nanosheets.

To further improve the electrochemical activity, ECT method is adopted. The ECT of O‐NFS ultrathin nanosheets is conducted by in situ negative linear sweeps from 0.126 to −0.274 V (vs RHE) for three times at a scan rate of 10 mV s^−1^ in 1 m KOH solution. Only 120 s is needed to complete the entire ECT process. Then, LSV curve of O‐NFS after ECT (denoted as O‐NFS‐ECT) is recorded (**Figure** **4**a). The required overpotential for reaching a current density of 200 mA cm^−2^ shifts negatively from 312 mV for O‐NFS to 294 mV for O‐NFS‐ECT. Obviously, the O‐NFS‐ECT reveals lower overpotentials at the same current densities compared with those of O‐NFS, confirming the superior OER activity of O‐NFS‐ECT. To investigate the ECT time effect, the different treatment times are set as 0, 80, 120, and 280 s (Figure S7, Supporting Information). It is found that the OER activity can be enhanced with the treatment time increased in the range of 0–120 s, however, it does not change when the treatment time is extended to much longer (280 s). Since the electrochemical activities are sensitive to the surface electronic states and active areas, it is necessary to measure XPS and electrochemical double‐layer capacitance (*C*_dl_) in proportion to the electrochemically active surface area (ECSA)^20^ to detect the surface changes before and after ECT. Figure 4b shows the Ni 2*p* XPS spectra of O‐NFS and O‐NFS‐ECT. The Ni^2+^ peak shows a negative shift (≈0.2 eV) compared to that of O‐NFS, when O‐NFS are electrochemically reduced by ECT. The electronic structure changes happen to Ni in O‐NFS‐ECT may weaken Ni—O bond from Ni‐OO species in the last step of OER process and thus accelerate the release of O_2_.^21^ The oxygen concentration of O‐NFS after ECT is calculated to be 3.0%, which is almost same to that of O‐NFS before ECT, indicating that the ECT process has no effect upon the oxygen concentration (Figure S8, Supporting Information). In addition, the *C*_dl_ of O‐NFS and O‐NFS‐ECT ultrathin nanosheets can be determined from the cyclic voltammetry (CV) curves, which were tested at different scan rates from 20 to 120 mV s^−1^ (Figure 4c,d). The O‐NFS‐ECT affords a larger active area than O‐NFS due to the increased CV area (Figure 4e), as evidenced by calculating *C*_dl_ of O‐NFS (0.4 mF cm^−2^) and O‐NFS‐ECT (1.1 mF cm^−2^) ultrathin nanosheets (Figure 4f). To understand and Figure out the synergetic role of electronic structure and ECSA, we normalize the current densities of O‐NFS and O‐NFS‐ECT by their corresponding relative ECSA (Figure S9, Supporting Information). The improvement effect of catalytic activity after the relative ECSA normalization is not as obvious as that after the geometric electrode area normalization, highlighting the advantage of ECSA in enhancing catalytic activity. As a result, the ECT can effectively regulate the electronic structure and increase the active sites, giving a reasonable explanation for the enhanced OER activity.

**Figure 4:**
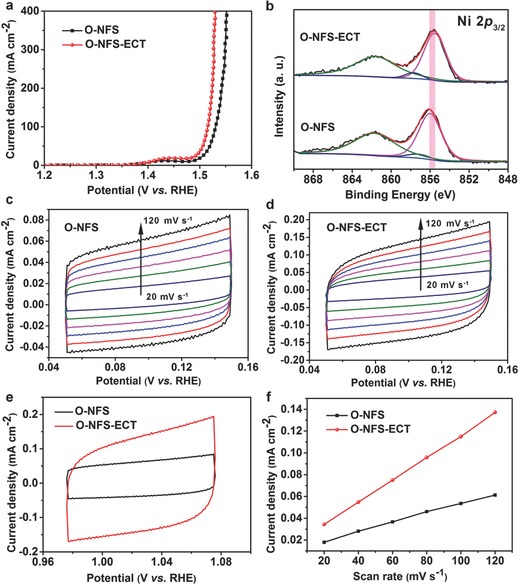
a) LSV curves of O‐NFS and O‐NFS‐ECT. b) Ni 2*p* XPS spectra of O‐NFS and O‐NFS‐ECT ultrathin nanosheets. Cyclic voltammetry (CV) curves of c) O‐NFS and d) O‐NFS‐ECT ultrathin nanosheets measured at different scan rates from 20 to 120 mV s^−1^. e) CVs of O‐NFS and O‐NFS‐ECT ultrathin nanosheets at a scan rate of 120 mV s^−1^. f) Plots of the current density at 1.026 V vs scan rate for O‐NFS and O‐NFS‐ECT ultrathin nanosheets.

The O‐NFS‐ECT as the optimized catalyst is conducted to probe the OER application potentials. For comparison, RuO_2_ with the same mass loading of 0.17 mg cm^−2^ deposited on Ti plate, and bare Ti plate are also measured (**Figure** **5**). As expected, the O‐NFS‐ECT exhibits the best OER performance (Figure 5a). It needs a very low overpotential of 259 mV at a current density of 20 mA cm^−2^, much smaller than that of RuO_2_ (370 mV). Note that, the O‐NFS‐ECT achieves a large current density up to 500 mA cm^−2^ under an extremely low overpotential of 300 mV, which can meet the demand for industrial applications.[[qv: 2a]] The corresponding Tafel slopes for O‐NFS‐ECT and RuO_2_ are calculated as 39 and 71 mV dec^−1^, respectively (Figure 5b). Nyquist impedance plots (Figure S10, Supporting Information) of O‐NFS‐ECT and RuO_2_ show that the O‐NFS‐ECT possesses a lower charge‐transfer resistance compared with that of RuO_2_. The smaller Tafel slope and charge‐transfer resistance of O‐NFS‐ECT implies the faster reaction kinetics.^22^ The turnover frequency (TOF) is calculated to assess the intrinsic OER activities of O‐NFS‐ECT.^23^ The TOF value at the overpotential of 300 mV is estimated to be 0.76 s^−1^. We further examine the electrochemical stability of O‐NFS‐ECT by chronopotentiometric curve and LSV curves before and after 1000 cycles, as shown in Figure 5c and its inset. The results reveal the excellent performance of O‐NFS‐ECT keep unchanged without degradation, even under the condition of incessant high current density of 165 mA cm^−2^ and vigorous bubbles surging. When applied higher potentials, the O‐NFS‐ECT catalyst can deliver much larger current density up to 3000 mA cm^−2^ at an overpotential of 435 mV and remain the constant current density of 1100 mA cm^−2^ for 12 h (Figure 5d and its inset). Surprisingly, the OER activity can be further improved when O‐NFS‐ECT catalyst is deposited on Au‐coated Ti plate (O‐NFS‐ECT/Au‐Ti). The overpotential at 2000 mA cm^−2^ decreases from 362 mV for O‐NFS‐ECT/Ti to 326 mV for O‐NFS‐ECT/Au‐Ti, making it the best OER activity yet reported. The improved performance may be ascribed to the highly active O‐NFS‐ECT and Au interfacial sites.[[qv: 5b]] More importantly, the O‐NFS‐ECT exhibits 100% Faradic efficiency for OER (**Figure** **6**). Considering the lower overpotential at high current density, small Tafel slope and high TOF, the O‐NFS‐ECT catalyst undoubtedly has the preeminent OER activity compared with many other Ni‐based catalysts (Table S1, Supporting Information). In addition, the morphology of O‐NFS‐ECT after a series of OER measurements is characterized (Figure S11, Supporting Information). The SEM and TEM images indicate the O‐NFS‐ECT catalyst still maintains the original ultrathin nanosheets architecture, further demonstrating the robust and strong structural stability of O‐NFS‐ECT. Such outstanding OER performance can be attributed to the following reasons: (1) the self‐supported 3D open architecture composed of amorphous‐like ultrathin nanosheets, endowing them with more active sites, efficient charge and mass transport, and structural stability for electrocatalysis. (2) the synergistic regulations of electronic structure and catalytically active areas by combining the oxygen‐incorporation and ECT process.

**Figure 5:**
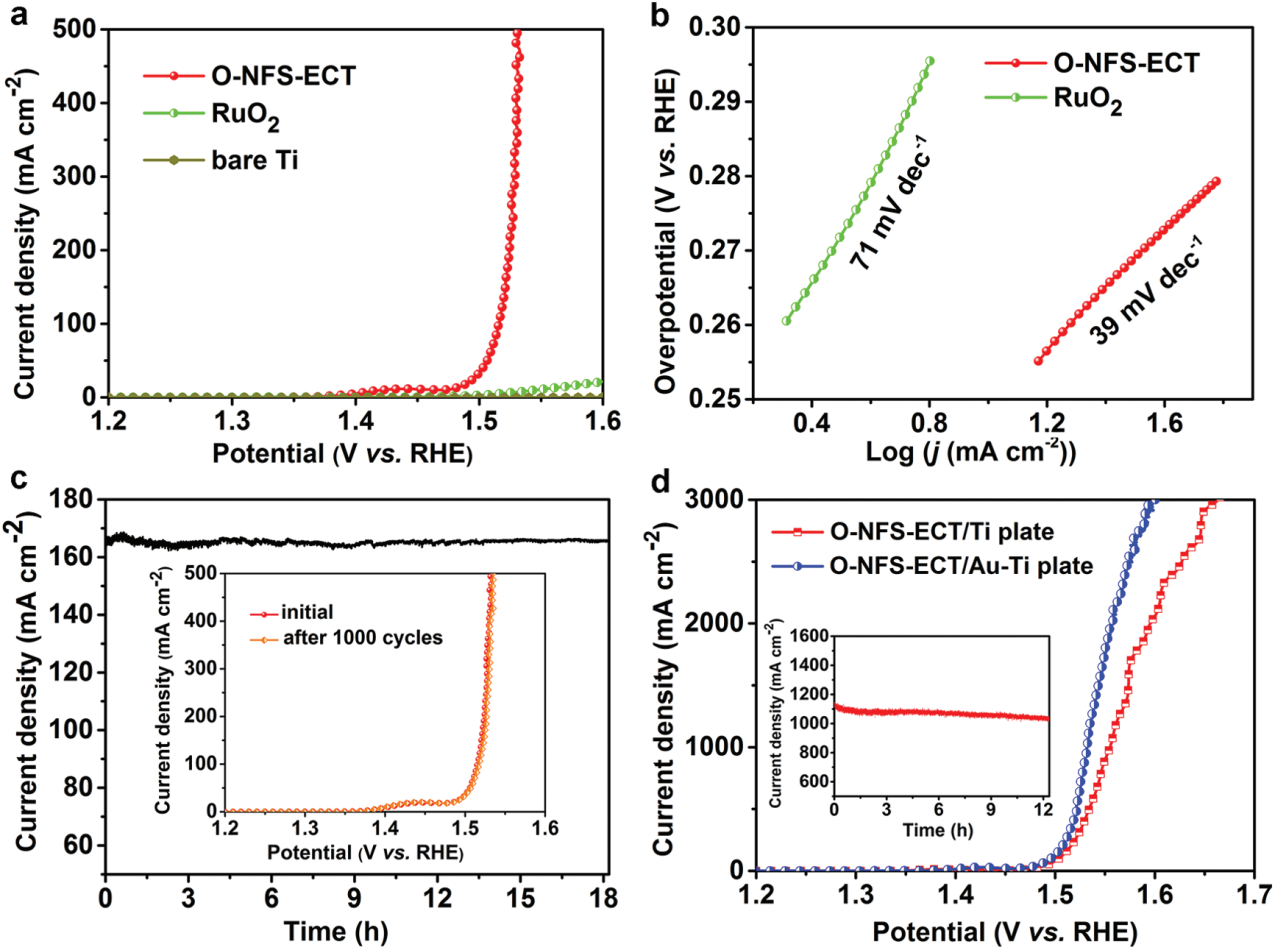
a) LSV curves of O‐NFS‐ECT, RuO_2_ and bare Ti plate. b) Tafel plots of O‐NFS‐ECT and RuO_2_. c) Chronopotentiometric curve of O‐NFS‐ECT with a constant current density of 165 mA cm^−2^. Inset in c): LSV curves of initial O‐NFS‐ECT and after 1000 CV cycles. d) LSV curves of O‐NFS‐ECT deposited on pure Ti plate and Au‐coated Ti plate at high applied potentials. Inset in d): chronopotentiometric curve of O‐NFS‐ECT with a constant current density of 1100 mA cm^−2^.

**Figure 6:**
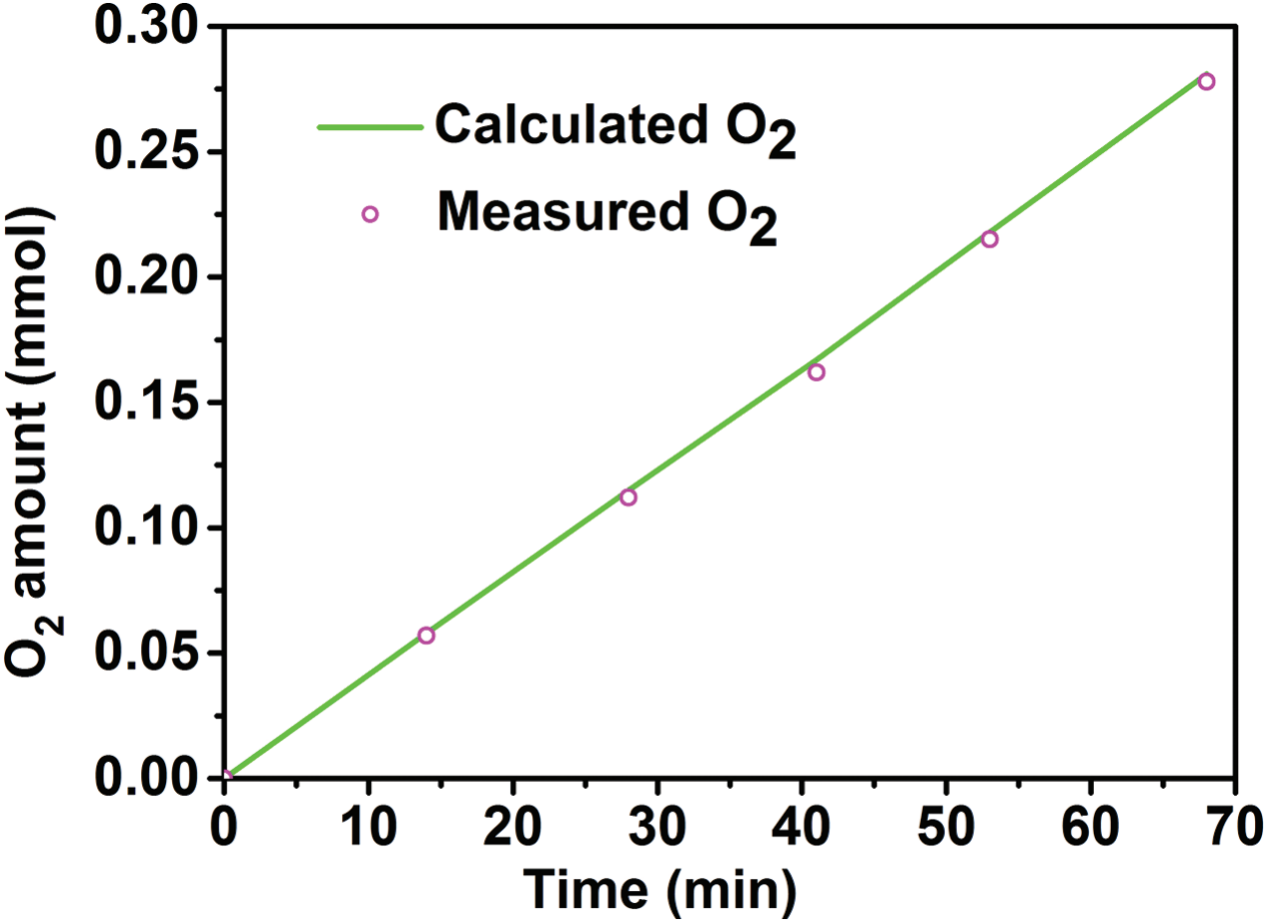
The amount of O_2_ theoretically calculated and experimentally measured versus time for the OER of O‐NFS‐ECT.

In summary, we demonstrate a facile strategy to synthesize O‐NFS ultrathin nanosheets by calcination of NFS, followed by a fast ECT process (120 s) as an efficient OER catalyst, especially at large current densities. The strategy combining the oxygen incorporation with ECT successfully realizes the synergistic regulations of electronic and structural (active sites) properties. The influence of oxygen‐incorporation and ECT on the OER activity is also investigated. The O‐NFS‐ECT catalyst offers new possibilities to meet the industrial demand of highly efficient catalysts. It shows large current densities of 500 and 3000 mA cm^−2^ at low overpotentials of 300 and 435 mV, a small Tafel slope of 39 mV dec^−1^, a high TOF value of 0.76 s^−1^, and outstanding stability at 1100 mA cm^−2^ for 12 h in 1 m KOH solution. The excellent performance can be ascribed to the unique 3D open architecture consisted of amorphous‐like ultrathin nanosheets with more exposure of active sites and improved conductivity, as well as the proper electronic structure benefiting from the oxygen incorporation and ECT. More importantly, the work may pave a new avenue to design other electrocatalysts with high performance and stability toward energy conversion and storage applications.

## Experimental Section

*Preparation of Ni*–*Fe–S Ultrathin Nanosheets*: NFS ultrathin nanosheets were prepared by an electrodeposition method in a standard three‐electrode electrochemical cell with Ti plate, Pt plate, and saturated calomel reference electrode (SCE) as the working electrode, counter electrode, and reference electrode, respectively. The electrolyte bath contained 4 × 10^−3^ m Ni(NO_3_)_2_·6H_2_O, 1.2 × 10^−3^ m Fe(NO_3_)_3_·9H_2_O and 0.5 m thiourea. The constant potential electrodeposition was carried out at −1.044 V (vs SCE) for 11 min. The electrodeposited Ti plate was cleaned by rinsing with water and ethanol, followed by drying in air at room temperature. The mass loading of NFS ultrathin nanosheets on Ti plate was determined by the mass differences before and after the electrodeposition using a microbalance (Mettler Toledo XS205). The typical mass loading is about 0.17 mg cm^−2^.

*Preparation of Ni–S or Fe–S Ultrathin Nanosheets*: The preparation procedures of Ni–S or Fe–S ultrathin nanosheets were similar to the preparation of NFS ultrathin nanosheets, except that 4 × 10^−3^ m Ni(NO_3_)_2_·6H_2_O and 1.2 × 10^−3^ m Fe(NO_3_)_3_·9H_2_O were replaced with 5.2 × 10^−3^ m Ni(NO_3_)_2_·6H_2_O or 5.2 × 10^−3^ m Fe(NO_3_)_3_·9H_2_O.

*Preparation of Oxygen‐Incorporated Ni–Fe–S Ultrathin Nanosheets*: The oxygen‐incorporated ultrathin nanosheets were obtained by annealing the electrodeposited NFS ultrathin nanosheets at 200 °C for 1 and 5 h in air (denoted as O‐NFS and O‐NFS‐con, respectively).

*Electrochemical Tuning of Oxygen‐Incorporated Ni–Fe–S (O‐NFS‐ECT) Ultrathin Nanosheets*: The electrochemical tuning of O‐NFS was conducted in 1 m KOH electrolyte by in situ negative linear sweeps from 0.126 to −0.274 V (vs RHE) for three times at a scan rate of 10 mV s^−1^. Only 120 s is needed to complete the entire ECT process. Then, the O‐NFS‐ECT ultrathin nanosheets were obtained.

*Structural Characterization*: The SEM images were taken with a Hitachi S‐4800 scanning electron microscope. TEM and HRTEM images were obtained with FEI Tecnai G2 F20 system equipped with GIF 863 Tridiem (Gatan), and EDS elemental distribution images were determined by JEM 2100F transmission electron microscope. The XRD was recorded with a Bruker D8 Focus Diffraction System using a Cu Kα source (λ = 0.154178 nm). Raman spectra were recorded on a RENISHAW inVia reflex Raman Microscope at excitation laser wavelength of 532 nm. XPS analysis was performed on a PHI 5000 Versaprobe system using monochromatic Al Kα radiation. All binding energies were referenced to the C 1*s* peak at 284.8 eV.

*Electrochemical Measurements*: Electrochemical measurements were carried out in a typical three‐electrode cell consisting of a working electrode, a glassy carbon counter electrode, and a Hg/HgO (1 m KOH) reference electrode using an electrochemical workstation (CHI 660D, CH Instruments, Austin, TX). The Ni–Fe–S catalyst samples on Ti plate were directly used as the working electrode. The RuO_2_ sample as control catalyst was dispersed in water/isopropanol with Nafion solution and drop‐cast into the Ti plate. All the loading mass of the catalysts on the Ti plate was about 0.17 mg cm^−2^. All the potentials in the text, if not specified, were recorded relative to the reversible hydrogen electrode (vs RHE) and the current density was normalized to the effective geometrical surface area. OER measurements were carried out in the presence of Ar‐saturated 1 m KOH (pH = 14.00) as electrolyte. The electrochemical impedance spectroscopy measurements were carried out in the same configuration at 1.576 V (vs RHE) from 1000 KHz to 1 Hz. The Faradaic efficiency was calculated by comparing the amount of gas theoretically calculated and experimentally measured. The gas experimentally generated from the water splitting was collected by water–gas displacing method. The theoretical amount of O_2_ was calculated by applying the Faraday law.

*TOF Calculation*: TOF was calculated according to the following Equation (1)

(1) $$\mathrm{TOF}=\frac{j\times A}{4\times F\times n}$$

where *j* is the measured geometrical current density at a given overpotential of 300 mV, *A* is the surface area of the electrode, the number 4 represents four electron transfer for per mole of O_2_, *F* is the Faraday constant, and *n* is the number of moles of the Ni atom on the electrode. The Ni content was calculated from the total charge by integration of the Ni^2+^/Ni^3+^ redox peak area in the cyclic voltammogram.

## Supporting information

As a service to our authors and readers, this journal provides supporting information supplied by the authors. Such materials are peer reviewed and may be re‐organized for online delivery, but are not copy‐edited or typeset. Technical support issues arising from supporting information (other than missing files) should be addressed to the authors.

Supplementary
