# Supplementary material for: Enhancing Oxygen Evolution Reaction at High Current Densities on Amorphous‐Like Ni–Fe–S Ultrathin Nanosheets via Oxygen Incorporation and Electrochemical Tuning
Source: Adv Sci (Weinh). 2016 Dec 20;4(3):1600343. doi: 10.1002/advs.201600343 (PMC5357987; doi:10.1002/advs.201600343)
Supplement: Supplementary file 1 — Supplementary [file ADVS-4-na-s001.pdf]

Copyright WILEY-VCH Verlag GmbH & Co. KGaA, 69469 Weinheim, Germany, 2016.

Supporting Information

for *Adv. Sci.,* DOI: 10.1002/advs.201600343

Enhancing Oxygen Evolution Reaction at High Current Densities on Amorphous-Like Ni–Fe–S Ultrathin Nanosheets via Oxygen Incorporation and Electrochemical Tuning

*Jingfang Zhang, Yuchen Hu, Dali Liu, Yu Yu, and Bin Zhang**

Copyright WILEY-VCH Verlag GmbH & Co. KGaA, 69469 Weinheim, Germany, 2013.

Supporting Information

Enhancing Oxygen Evolution Reaction at High Current Densities on Amorphous-Like Ni-Fe-S Ultrathin Nanosheets via Oxygen Incorporation and Electrochemical Tuning

*Jingfang Zhang, Yuchen Hu, Dali Liu, Yu Yu, and Bin Zhang**

Morphology characterization of NFS

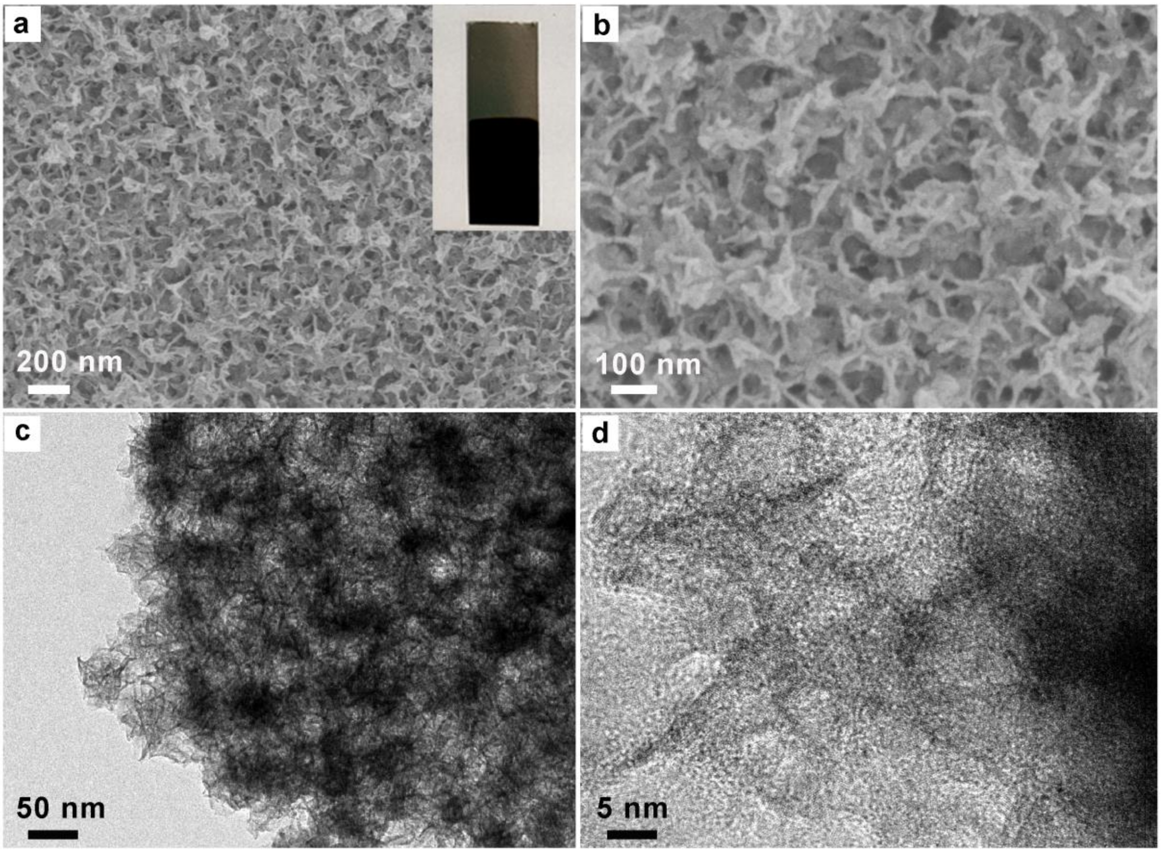

Figure S1. (a) Low- and (b) high-magnification SEM images of as-prepared NFS samples. Inset in (a) is the photograph of the as-prepared NFS samples on Ti plate. (c) A typical TEM image of NFS samples. (d) HRTEM image of NFS samples.

XPS spectra of Ni 2$p_{3/2}$, Fe 2$p_{3/2}$, S 2p regions for NFS ultrathin nanosheets

X-ray photoelectron spectra (XPS) were used to characterize the surface structure of NFS ultrathin nanosheets. As shown in Figure S2, the Ni 2p peaks are dominated by Ni^{2+} and a small proportion of Ni^{3+} exists in NFS nanosheets. The high resolution Fe 2p region shows characteristic peaks of the Fe^{2+} (713.5 eV) and Fe^{3+} (711.1 eV). The S 2p spectrum reveals the existence of S^{2-} and oxidation state in the NFS ultrathin nanosheets.

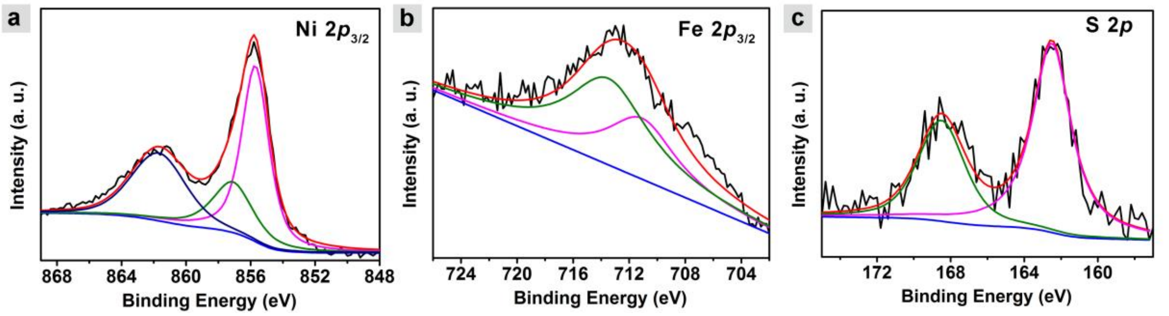

Figure S2. XPS spectra of high resolution (a) Ni 2$p_{3/2}$, (b) Fe 2$p_{3/2}$, and (c) S 2p regions for NFS.

XRD pattern of O-NFS ultrathin nanosheets

All the typical diffraction peaks are associated with Ti (JCPDS Card No. 44-1294) and no other diffraction peaks can be seen, revealing the amorphous nature of O-NFS ultrathin nanosheets.

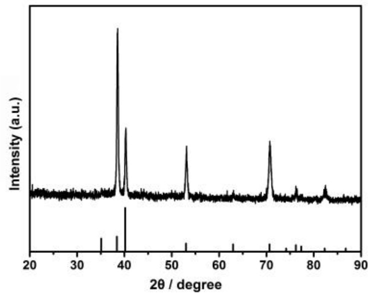

Figure S3. XRD pattern of O-NFS ultrathin nanosheets.

XPS spectra of Ni 2$p_{3/2}$, Fe 2$p_{3/2}$, S 2p regions for O-NFS ultrathin nanosheets

Figure S4a shows the Ni 2$p_{3/2}$ region of O-NFS. The binding energies at 855.9 and 857.2 eV correspond to Ni^{2+} and Ni^{3+}, respectively. The satellite peak at 861.7 eV is shakeup peak of Ni 2$p_{3/2}$.[1] In the Fe 2$p_{3/2}$ region (Figure S4b), the peak at binding energy of 711.4 eV is ascribed to Fe^{3+}.[2] In the S 2p XPS spectrum (Figure S4c), the peaks located at binding energies of 162.1 and 168.4 eV are indexed to S^{2-} and surface oxidized sulfur species, respectively.[3,4]

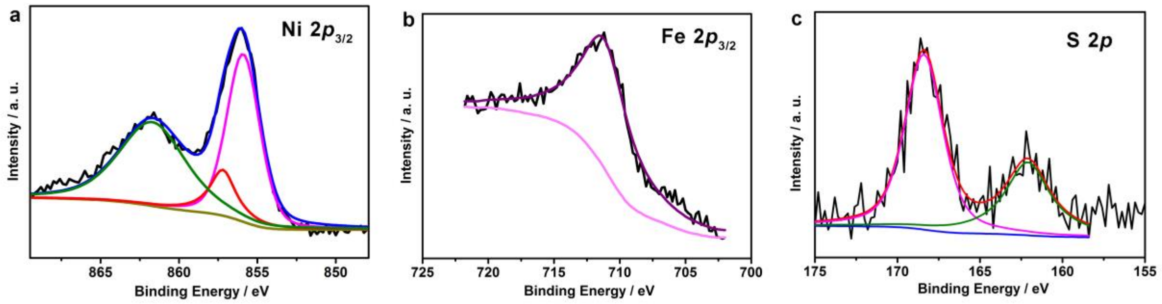

Figure S4. XPS spectra of high resolution (a) Ni 2$p_{3/2}$, (b) Fe 2$p_{3/2}$, and (c) S 2p regions for O-NFS.

OER activity of Ni-S and Fe-S samples

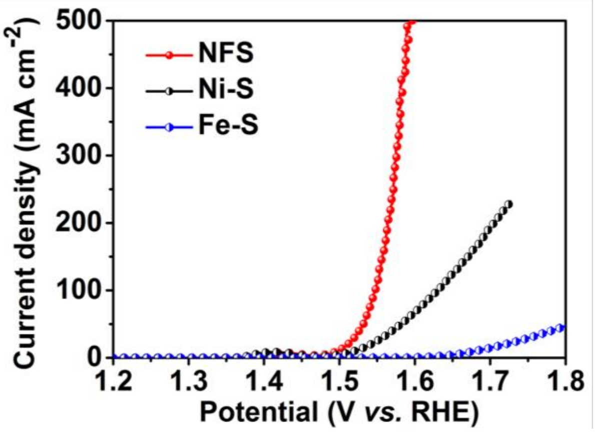

Figure S5. LSV curves of Ni-S, Fe-S and NFS samples.

XPS spectrum and OER activity of O-NFS and O-NFS-con

The oxygen amount in the form of metal-oxygen bonds was calculated as 5.5 % based on the XPS analysis. The O-NFS has better OER performance than O-NFS-con with larger oxygen incorporation amount (5.5 %), suggesting the proper oxygen-incorporation amount is important for the OER activity.

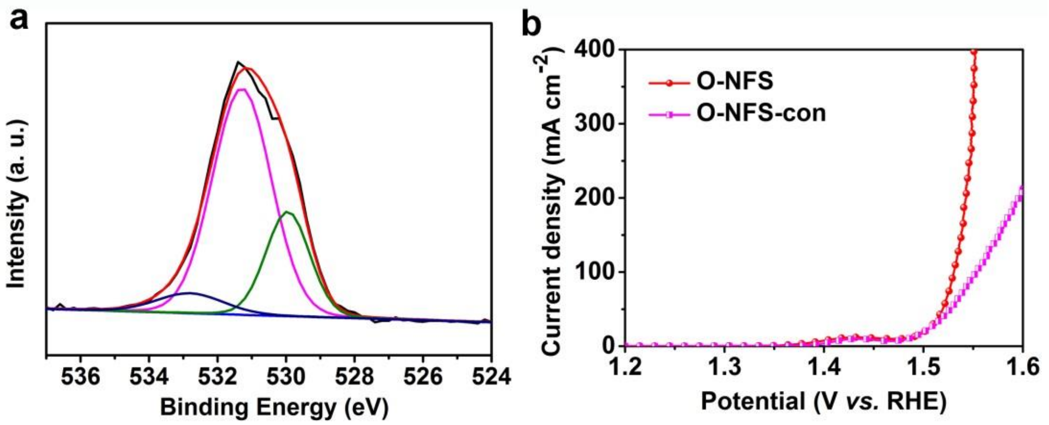

Figure S6. (a) O 1s XPS spectrum of O-NFS-con ultrathin nanosheets. (b) LSV curves of O-NFS and O-NFS-con ultrathin nanosheets in 1 M KOH.

OER activity of O-NFS with different ECT treatment time.

To investigate the ECT time effect, the different treatment times are set as 0s, 80s, 120s and 280s. The obtained samples are termed as O-NFS-ECT-x, where x represents treatment time. It is found that the OER activity can be enhanced with the treatment time increased in the range of 0-120s, however, it does not change when the treatment time is extended to much longer (280s).

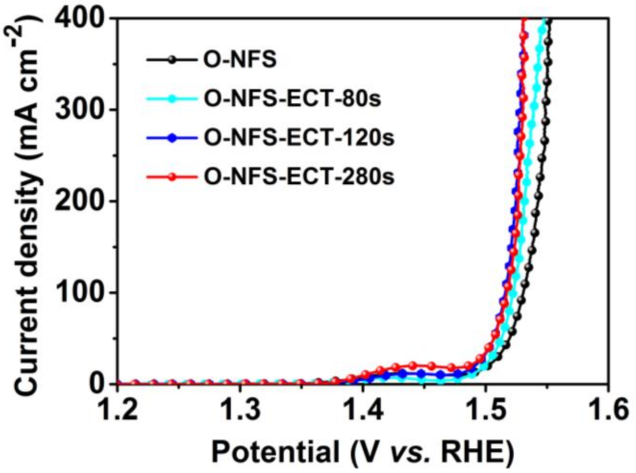

Figure S7. LSV curves for O-NFS with different ECT treatment time.

XPS spectra of O-NFS-ECT

The oxygen concentration of O-NFS after ECT is calculated to be 3.0 % based on the XPS analysis. The value is almost same with that of O-NFS before ECT, indicating that the ECT process has no effect upon the oxygen concentration. The binding energies of Fe 2p and S 2p of O-NFS after ECT have no obvious shift and chemical state change, suggesting that the ECT process only works for Ni rather than Fe and S.

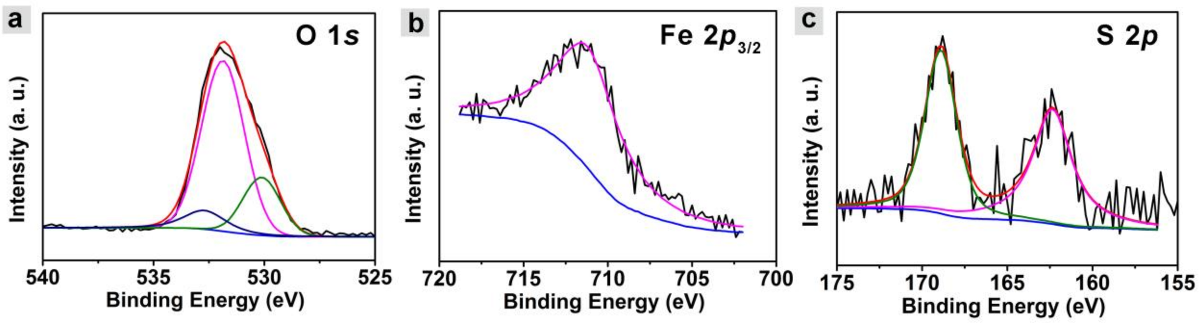

Figure S8. XPS spectra of (a) O 1s, (b) Fe 2$p_{3/2}$, and (c) S 2p regions of O-NFS-ECT ultrathin nanosheets.

OER activity of O-NFS and O-NFS-ECT by relative ECSA normalization.

Because the C_{dl} is proportional to the electrochemically active surface area (ECSA) of electrocatalyst, we use the ECSA of O-NFS as the benchmark and set the relative ECSA of O-NFS as 1.00, so the relative ECSA of O-NFS-ECT is 2.75. After the relative ECSA normalization, the specific activity of O-NFS-ECT is 77.8 mA cm^{-2}_{ECSA} at an overpotential of 300 mV, which is 1.5 times than that of O-NFS (50.6 mA cm^{-2}_{ECSA}). The improvement effect of catalytic activity after the relative ECSA normalization is not as obvious as that after the geometric electrode area normalization, highlighting the advantage of ECSA in enhancing catalytic activity.

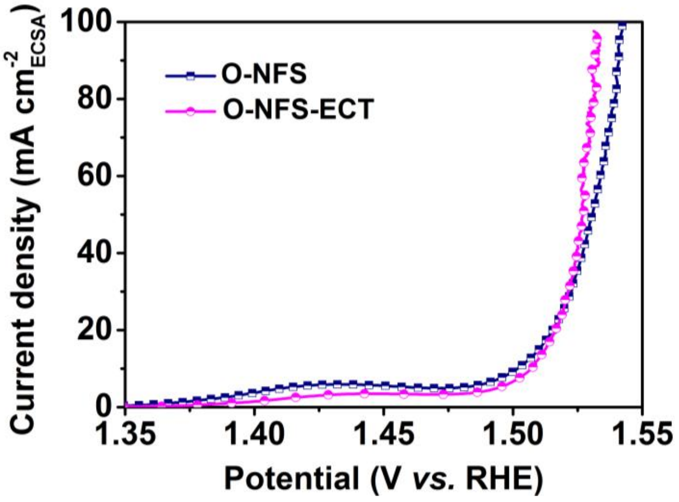

Figure S9. LSV curves for O-NFS and O-NFS-ECT normalized by the relative electrochemically active surface area (ECSA).

EIS measurement

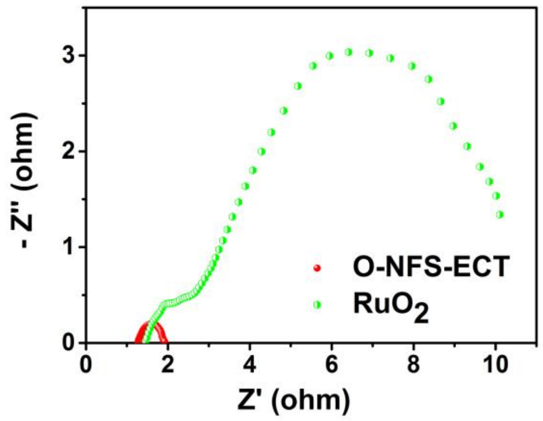

Figure S10. Nyquist plots of the O-NFS-ECT and RuO_2 at 1.576 V *vs.* RHE.

Morphology characterization of O-NFS-ECT after the OER

The SEM and TEM images suggest that the O-NFS-ECT electrocatalyst still maintains the original ultrathin nanosheet architecture after OER measurements.

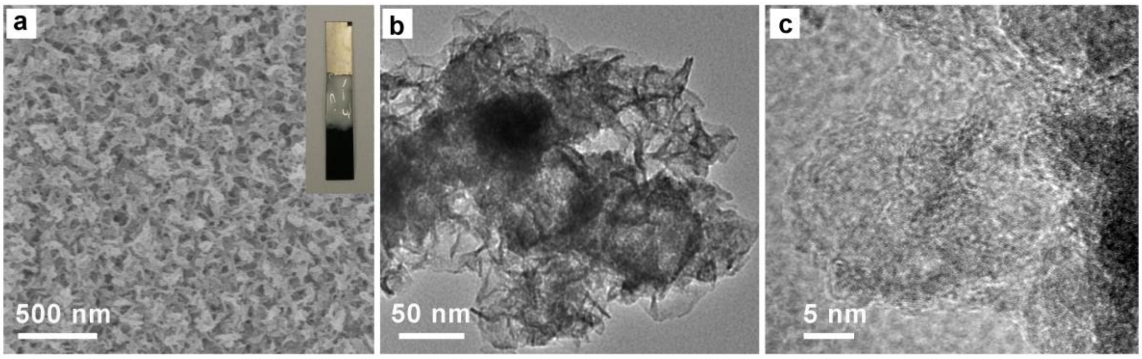

Figure S11. (a) SEM, (b) TEM and (c) HRTEM images of O-NFS-ECT after a series of OER tests. Inset in (a) is the photograph of the catalyst electrode.

Table S1. Comparison of the OER activity of Ni-based catalysts in 1 M KOH recently reported.

Sample	Catalyst loading (mg cm^{-2})	Current density (j) / mA cm^{-2}	Overpotential (η) / mV at the corresponding j	Tafel slope / mV dec^{-1}	TOF / s^{-1}	Ref.
**O-NFS-ECT**	**0.17**	**500**<br>**3000**	**300**<br>**435**	**39**	**0.76 at η = 300 mV**	**This work**
NiS_x/Ni foam	–	500	316	96	–	3
Ni_3FeN	0.35	10<br>900	280<br>970	46	–	5
Ni_3C/C	0.285	140	400	46	0.099 at η = 387 mV	6
NiFe LDH/ carbon nanotube	0.2	10	247	31	0.56 at η = 300 mV	7
NiFe LDH	0.07	10	> 300	40	0.24 at η = 300 mV	8
FeNi-rGO LDH/Ni foam	0.25	10	206	39	0.98 at η = 300 mV	9
NiSe/Ni foam	2.8	20<br>350	270<br>~ 370	64	–	10
NiP/Ni foam	–	30<br>100	270<br>~ 320	73.2	0.1 at η = 300 mV	11

Reference

(1) Lu, X.-F.; Wu, D.-J.; Li, R.-Z.; Li, Q.; Ye, S.-H.; Tong, Y.-X.; Li, G.-R. *J. Mater. Chem. A* **2014**, *2*, 4706.

(2) Feng, J.-X.; Xu, H.; Dong, Y.-T.; Ye, S.-H.; Tong, Y.-X.; Li, G.-R. *Angew. Chem. Int. Ed.* **2016**, *55*, 3694.

(3) You, B.; Sun, Y. *Adv. Energy Mater.* **2016**, *6*, 1502333.

(4) Ge, X.; Chen, L.; Zhang, L.; Wen, Y.; Hirata, A.; Chen, M. *Adv. Mater.* **2014**, *26*, 3100.

(5) Jia, X.; Zhao, Y.; Chen, G.; Shang, L.; Shi, R.; Kang, X.; Waterhouse, G. I. N.; Wu, L.-Z.; Tung, C.-H.; Zhang, T. *Adv. Energy Mater.* **2016**, *6*, 1502585.

(6) Xu, K.; Ding, H.; Lv, H.; Chen, P.; Lu, X.; Cheng, H.; Zhou, T.; Liu, S.; Wu, X.; Wu, C.; Xie, Y. *Adv. Mater.* **2016**, *28*, 3326.

(7) Gong, M.; Li, Y.; Wang, H.; Liang, Y.; Wu, J. Z.; Zhou, J.; Wang, J.; Regier, T.; Wei, F.; Dai, H. *J. Am. Chem. Soc.* **2013**, *135*, 8452.

(8) Song, F.; Hu, X. *Nat. Commun.* **2014**, *5*, 4477.

(9) Long, X.; Li, J.; Xiao, S.; Yan, K.; Wang, Z.; Chen, H.; Yang, S. *Angew. Chem. Int. Ed.* **2014**, *53*, 7584.

(10) Tang, C.; Cheng, N.; Pu, Z.; Xing, W.; Sun, X. *Angew. Chem. Int. Ed.* **2015**, *54*, 9351.

(11) Chen, G.-F.; Ma, T. Y.; Liu, Z.-Q.; Li, N.; Su, Y.-Z.; Davey, K.; Qiao, S.-Z. *Adv. Funct. Mater.* **2016**, *26*, 3314.
